# Synthetic Biology and Conservation of Nature: Wicked Problems and Wicked Solutions

**DOI:** 10.1371/journal.pbio.1001530

**Published:** 2013-04-02

**Authors:** Kent H. Redford, William Adams, Georgina M. Mace

**Affiliations:** 1 Archipelago Consulting, Irvington, New York, United States of America; 2 Department of Geography, University of Cambridge, Cambridge, United Kingdom; 3 Centre for Biodiversity and Environment Research, GEE, University College London, London, United Kingdom

## Abstract

So far, conservation scientists have paid little attention to synthetic biology; this is unfortunate as the technology is likely to transform the operating space within which conservation functions, and therefore the prospects for maintaining biodiversity into the future.

Extinction might not be forever if synthetic biologists and others pursue their proposals to use advanced genetic engineering techniques to save endangered species and return extinct ones [1]. This is only the most eye-catching example of a broad engagement that will soon take place between the synthetic biology community and the biodiversity conservation community that may change the relationship between humans and the natural world. Though these communities are strangers to each other now, the work they do and the goals they pursue are in places complementary and in others conflicting but uninformed by each other. A respectful and open discussion between these two communities and society at large is urgent to determine how to proceed on issues that overlap.

Recent international and intergovernmental meetings of the Convention on Biodiversity (CBD) (October 2012) and the International Union for Conservation of Nature (September 2012) have reconfirmed the serious attention the world community is paying to the vital need to conserve the natural world. Commitments made by 94 governments in April 2012 to establish the Intergovernmental Platform on Biodiversity and Ecosystem Services (IPBES) are a tangible response to addressing the ever-increasing threats to global biodiversity, against the approaching beat of a changing climate. These threats have created a set of “wicked problems” [2], that are messy, intractable, subject to multiple interpretations, and for which solutions at present are not evident or inscrutable. Dealing with the causes and consequences of biodiversity loss in a changing environment is one such problem.

Over the past century an array of conservation strategies of increasing sophistication and scope have emerged to address biodiversity loss [3]. The Aichi Biodiversity Strategy, adopted in 2010 by the Conference of the Parties (COP) to the CBD, distills these strategies into a set of 20 targets adopted by the international community. The Strategy is designed to a) address the underlying causes of biodiversity loss by mainstreaming biodiversity across government and society; b) reduce the direct pressures on biodiversity and promote sustainable use; c) improve the status of biodiversity by safeguarding ecosystems, species, and genetic diversity; d) enhance the benefits to all from biodiversity and ecosystem services; and e) enhance implementation through participatory planning, knowledge management, and capacity building [4].

The Aichi Targets and Strategic Goals are challenging and will require the full array of tools, techniques and approaches if progress is to be made. Yet, to date both the targets, and the institutional arrangements that support them, are built on an understanding of biodiversity and ideas about conservation strategies developed over the twentieth century, and have barely considered new scientific and engineering prospects such as those found in the emerging field of synthetic biology.

The Presidential Commission for the Study of Bioethical Issues [5] defined synthetic biology as “a scientific discipline that relies on chemically synthesized DNA, along with standardized and automatable processes, to address human needs by the creation of organisms with novel or enhanced characteristics or traits.” The field is moving fast [6],[7]. Billions of dollars are being invested globally, and developments of novel applications or improvements of existing ones emerge weekly [8],[9]. Huge claims are routinely made about the potential benefits of synthetic biology: “many of the major global problems, such as famine, disease and energy shortages, have potential solutions in the world of engineered cells” [10]. Lloyds of London's Emerging Risk Group wrote in 2009: “Many believe that Synthetic Biology will be one of the transformative technologies necessary to combat climate change, energy shortages, food security issues and water deficits” [11].

Synthetic biology has the potential to transform many aspects of human economy and society, and the environment, not least as a key technology in an emerging “bioeconomy”. Citing the impacts of existing biofuel production, some are deeply suspicious of its possible impacts: the ETC Group suggests that “The proposed use of synthetic microbes in the production of the next generation of fuels, medicines and industrial chemicals may massively increase human impact on biodiversity, while accelerating biopiracy and making a mockery of any notion of ‘benefit sharing’” [12]. As Marris and Rose [13] observe, when discussing synthetic biology, “utopias and dystopias seem to be the only scenarios possible.”

Despite growing general debate, there has been surprisingly limited consideration of the risks or advantages of synthetic organisms to the conservation of biological diversity [14]. In the seven years prior to 2011, some 40 reports (in the English language alone) were published addressing the social, ethical, and legal issues raised by synthetic biology [15]. Ethicists and philosophers have considered the ways synthetic biology may change the relationship between humans and the natural world [16]–[18], and considerable discussion has taken place about who will be in control of synthetic biology (e.g., [6]). Critics have identified potential dangers of genetically modified organisms on native species, the resilience of natural ecosystems, small-scale producers in developing countries, and public safety [19]–[22]. Horizon scanning studies have highlighted technologies that involve genetic engineering, such as the transfer of nitrogen-fixing ability to cereals [23].

However, thus far, conservation scientists appear to have paid little attention to synthetic biology as a field of science and technology. Recent surveys in biodiversity science have outlined many of the problems and promises that face the natural world [24]–[27], yet synthetic biology has gone virtually unnoticed. Noticed or not, change is coming, as the recently completed CBD COP 11 resolved to “…consider the potential positive and negative impacts of components, organisms and products resulting from synthetic biology techniques on the conservation and sustainable use of biodiversity”—but only to recommend further study [28].

The limited and timid engagement of conservation science and policy with the development of synthetic biology is unfortunate, because the technology is likely to transform the operating space within which conservation functions, and therefore the prospects for maintaining biodiversity into the future. The shape of this transformation is unclear. There are possibilities that synthetic biology may provide new solutions to established “wicked” problems such as disease affecting wildlife (e.g., [29]) and may alleviate pressure on ecosystems by supplying sustainable food for a future world of 9 billion people. There are also potentially negative impacts on natural and managed ecosystems and human welfare through the release of novel organisms [19]–[22]. The potential consequences for biodiversity conservation of even the promise of innovations from synthetic biology are significant. Considering the Aichi Targets (see above), we suggest (Table 1) some plausible consequences of synthetic biology advances for the way that targets are addressed, the side effects of assuming the techniques work, and ultimate impacts on the wild species and habitats for which the targets were devised.

**Table 1:** Examples of how synthetic biology, promised or developed at even modest scales, could significantly affect the Aichi Biodiversity Targets.

Aichi Strategic Goal	Examples of Potential Impact of Synthetic Biology
A. Address the underlying causes of biodiversity loss by mainstreaming biodiversity across government and society.(Targets 1–4)	• Peoples' awareness of biodiversity may be affected by an ability to artificially transform organism genomes, eroding understandings of what “nature” is (1)• Transition to sustainable production and consumption (which protects biodiversity) may be promoted (4)• Proposed synthetic biology solutions might move policymakers away from addressing underlying causes for biodiversity loss (4)
B. Reduce the direct pressures on biodiversity and promote sustainable use.(Targets 5–10)	• Synthetic traits in organisms may promote invasive capabilities (or novel organisms may be invasive) (9)• Synthetic organisms might improve potential for ecological restoration or creation (9)• The potential for synthetic organisms in the agricultural production sectors might foster “sustainable intensification” and “land sparing” to reduce land conversion and increase protection of wild habitats (6 and 7)• Industrial uses created by synthetic biology might drive significant land use change towards feedstock production (7 and 8)
C. To improve the status of biodiversity by safeguarding ecosystems, species, and genetic diversity.(Targets 11–13)	• Novel organisms might affect the integrity of protected areas (11)• Recreated extinct species may create credits to species lists, allowing natural species extinctions to occur while meeting targets to arrest species extinctions (12)• “Moral hazard” may reduce society's willingness to support measures to conserve endangered species (12)• Synthetic biology capability may make *ex situ* conservation more attractive relative to *in situ* with impacts on support for existing protected areas (13)
D. Enhance the benefits to all from biodiversity and ecosystem services.(Targets 14–16)	• Synthetic life forms could replace “nature's services” for clean water, clean air, etc., thereby removing the ecosystem services justification for nature conservation (14, 15)• Synthetic biology may extend private ownership of genetic material in ways that restrict access for public benefit (16)
E. Enhance implementation through participatory planning, knowledge management, and capacity building.(Targets 17–20)	• Since biological knowledge based on synthetic biology is both different and much more restricted than knowledge for biodiversity conservation, fundamental inequities may prevent the desired coherent, participatory actions for conservation (18 and 19)

There are 20 Targets grouped under five Strategic Goals agreed to by 193 countries that are Parties to the CBD in 2010. Individual target numbers are indicated in parentheses under each example. The full list of targets can be found at http://www.cbd.int/sp/targets/.

Conservation as a practice has frequently been backwards looking, focusing on reducing loss or on maintaining a status quo, an approach that has clearly not been effective in conserving biodiversity. Potential major shifts in the relationship between humans and nature such as those represented by synthetic biology would be better engaged with early and deeply. Yet of the hundreds of conservation practitioners with whom we have spoken, only a few had even heard of synthetic biology and had any sense of the changes it may bring. In order to expedite the engagement between the two fields, we have organized a meeting entitled “How will synthetic biology and conservation shape the future of nature?” to be held April 9–11, 2013, in Cambridge, United Kingdom (http://www.wcs.org/thefutureofnature). Our hope is that this meeting and this article will ultimately result in a practice of conservation educated about synthetic biology and a practice of synthetic biology educated about the concerns and imperatives of biodiversity conservation.

We do not know what will happen when synthetic biology practice meets conservation practice. There has been some speculation, but data cannot be gathered on what has not yet happened, leaving value-based claims free to proliferate. Yet it is imperative that conservation practitioners engage with synthetic biologists, not only to influence the practice to become “pro-conservation”, but also because without such informed engagement it would be too easy for policymakers and politicians to assume that synthetic biology solutions can provide easy fixes to intractable and expensive conservation actions and shift attention and support away from existing efforts such as protected area establishment and strengthening. Biodiversity, ecosystem services, and humans would all suffer from such decisions.

We suggest that conservation needs new thinking and new strategies to cope with the challenges of synthetic biology. We identify here five assertions that we believe highlight key emerging issues that need to be addressed by conservation scientists and practitioners, and institutions such as the CBD and IPBES.

- Extinction may not be forever. There are on-going attempts to recreate endangered species using the tools of synthetic biology. These include the woolly mammoth, the passenger pigeon, and the thylacine [30]. If successful, would such species be regarded as representatives of the species to which extinct forbears belonged? Or would they be viewed as “invasives from the past” and a threat to existing species? In accounting terms, how would extinction rates in conservation targets deal with recreated species? Currently such experiments are slow and hugely costly, but if such costs fall as some predict (by analogy with the costs and power of computing), such re-creations might become routine and affordable. How would choices be made about which species to save? More fundamentally, what conservation value would these forms have if the habitats that once supported them are gone? Might we face the moral hazard whereby confidence in our ability to recreate extinct species undermines our willingness to conserve naturally occurring biodiversity [31]?
- Synthetic life evolves. How will synthetic organisms interact with existing species and how far will such interactions be predictable from current ecological understanding of interspecific interactions? Will they become invasive and damage existing communities, or might they be safe and useful in restoring degraded or polluted ecosystems or address other ecological problems that have been intractable to date? Will the incorporation of synthetic organisms into ecosystems (e.g., through field agriculture, medical application, or accidental release) be seen as adding to the living diversity of the ecosystems in which they are incorporated and, if so, will these be judged as of higher value, or will loss of authenticity mean they are judged degraded [32]? Who will regulate the release of synthetic organisms outside the contained laboratory: will the permissive regulatory environment of “garage biology” be widely endorsed, will national governments try to establish individual regimes, and how will local and international views on the matter be taken into account?
- Our working definition of “natural” is no longer fit for purpose. Much of conservation is based on conserving ecosystems developed through ecological and evolutionary processes over the course of time, sometimes reflecting tight sets of inter-linkages that are hard to restore once lost. Will interactions between synthetic and natural organisms arise easily, or might the very different origins lead to largely disruptive impacts on natural communities? What would be the change to public perceptions of what is “natural” and the notion of evolution as a process beyond human construction? Will these technologies challenge the ethical basis for conservation action, as they have done in other settings [33]? How will we evaluate organisms created using novel nucleic acids as part of their genetic code—products of xenobiology [34]?
- Nature's services can be synthesized. The value of ecosystem to society is increasingly central to arguments about the importance of biodiversity [35]. One of the most common promises of synthetic biology is to engineer organisms that generate services of benefit to people (e.g., carbon sequestration, pollution control). What impact will this have on the relative value attached to natural ecosystems that already deliver these ecosystem services? Might ecosystems containing synthesized elements out-compete existing evolved ecosystems, delivering more services with less biodiversity?
- Synthetic life delivers private benefits. Many forms of life being developed by synthetic biology are being patented. The benefits provided by these organisms will reflect the economic interests of those able to invest in and develop them. This may well favor applications in existing industrial processes and commodity chains (energy, agriculture, aquaculture) and the operations of large business corporations. Impacts on the wider environment will tend to be treated as an externality. Knock-on impacts of price and other economic changes on smaller producers (e.g., smallholder farmers) will affect their decisions about land conversion and management, and hence future patterns of biodiversity loss. How will a balance be struck between private risk and gain versus public benefit and safety?

A serious need exists for wider discussion of the relationship between synthetic biology and biodiversity conservation, and what choices society can and should make. But this discussion is difficult, for two reasons. First, synthetic biology is a technical field little understood by non-experts. It will be difficult to create conditions for representative groups from society to engage in a well-informed, structured and balanced discussion. Second, these discussions are hard to frame because it is difficult to identify the right counterfactuals or alternative futures to compare with those underpinned by the new technology. It seems inevitable that synthetic biology will be a major factor in affecting the future. That future world will not be a slightly older version of the world that we currently inhabit. Rather, it will have a significantly altered climate, changed sea levels, novel pests and diseases, non-analog ecological communities, and a human population with changed priorities. The costs, benefits, and risks of synthetic biology need to be considered against that backdrop, not against a projected version of the present as is the common practice, but rather through mechanisms such as scenario development [36],[37]. This task is complicated by the fact that psychologists have shown how poor people are at thinking about the future—as Gilbert [38] has written, “because predictions about the future are made in the present, they are invariably influenced by the present.”

Synthetic biology brings with it a powerful attraction, causing biology to veer towards engineering with its inherent approach of human problem solving. It may prove to be a cure for certain wicked problems. But we suggest that now is the time to consider whether synthetic biology may be a wicked solution, creating problems of its own, some of which may be undesirable or even unacceptable in the area of biodiversity conservation.

But despite these difficulties, the discussion between conservation and synthetic biology must take place. It should not be based on alarmist or triumphalist positions, but on a clear-eyed examination of the norms, oversight, and public education necessary to make decisions about the enormous power of altering life on Earth. Such a careful, respectful, public discussion must examine the continuing role of conservation values. Much of conservation as currently practiced is predicated on the core ideals of wilderness and nature, though others envisage a carefully managed planet with all the biological components in place, albeit carefully tended by conscientious (human) custodians. Synthetic biologists propose to further equip humans to actively and consciously engineer the living world. The transformed world of 2050 will demand new strategies and new approaches in conservation.

## Abbreviations

CBD: Convention on Biodiversity
COP: Conference of the Parties
IPBES: Intergovernmental Platform on Biodiversity and Ecosystem Services
